# Serotonin regulates in a cell-type specific manner light-evoked response and synaptic activity in mouse retinal ganglion cells

**DOI:** 10.1186/s40659-025-00594-6

**Published:** 2025-03-04

**Authors:** Claudia Di Berardino, Sebastián F. Estay, Alejandro Alcaino, Andrés E. Chávez

**Affiliations:** 1 Programa de Doctorado en Neurociencias, Facultad de Ciencias, Universidad de Valparaíso, 2340000 Valparaíso, Chile; 2 Instituto de Neurociencias, Facultad de Ciencias, Universidad de Valparaíso, 2340000 Valparaíso, Chile; 3 Present Address: Stem Cell and Neurogenesis Unit, Division of Neuroscience, IRCCS San Raffaele Scientific Institute, 20132 Milan, Italy

**Keywords:** Serotonin transporter, Synaptic transmission, Retinal ganglion cells, Visual processing, Vision

## Abstract

**Background:**

Serotonin (5-HT) is known to be synthesized and accumulated in the vertebrate retina through the 5-HT transporter, SERT. While manipulation of the serotonergic system has been shown to impact visual processing, the role of 5-HT and SERT as modulators of retinal synaptic function remains poorly understood.

**Results:**

Using mouse retinal slices, we show that acute application of 5-HT produces a cell-type specific reduction in light-evoked excitatory responses (L-EPSC) in ON–OFF retinal ganglion cells (RGCs), but not in ON RGCs. Similarly, increasing 5-HT tone by acute application of citalopram, a selective 5-HT reuptake inhibitor, also reduces L-EPSC in ON–OFF RGCs while not affecting ON RGCs. Importantly, citalopram-mediated reduction of L-EPSC was absent in ON–OFF RGCs recorded from SERT null retina, highlighting the role of SERT in regulating light-evoked responses in RGCs. The effects of both exogenous and endogenous 5-HT on L-EPSC in ON–OFF RGCs are likely due to a presynaptic reduction in excitatory synaptic strength as 5-HT and citalopram reduced the frequency but not the amplitude of spontaneous excitatory currents (sEPSCs) in ON–OFF RGCs. Moreover, 5-HT and citalopram had no effect on currents elicited by the direct activation of postsynaptic receptors in RGCs by brief application of glutamate in the inner retina.

**Conclusions:**

Altogether these findings indicate that 5-HT modulates excitatory inputs onto RGCs in a cell-type specific manner and highlight that in the adult mouse retina, 5-HT-mediated effects onto RGCs are tightly controlled by the 5-HT transporter SERT.

**Supplementary Information:**

The online version contains supplementary material available at 10.1186/s40659-025-00594-6.

## Introduction

Serotonin (5-hydroxytryptamine, 5-HT), by regulating synaptic function and neuronal excitability throughout the central nervous system, plays an important role in cognitive and sensory functions [1, 2]. In the mammalian retina, all the components required for serotonergic regulation of neuronal function have been reported [3], including the enzymes necessary for 5-HT production (tryptophan hydroxylase, TPH) and degradation (monoamine oxidase, MAO), the vesicular monoamine transporter (VMAT2) required for its accumulation in synaptic vesicles, the 5-HT transporter (SERT) critical for its reuptake and depending on the species, including humans, different 5-HT receptor (5-HTR) subtypes distributed in distinct synaptic layers and cell types [3–11]. Accordingly, photoreceptors and amacrine cells can locally synthesize 5-HT, and a subset of bipolar and amacrine cells actively accumulate it [12–18]. Moreover, the evidence that the inner retina is innervated by serotonergic retinopetal axons originating in the dorsal raphe nuclei [19–22] that might also release 5-HT in the retinal circuit further underscores a role for the serotonergic system in retinal visual function. However, little is known about the cellular mechanisms underlying 5-HT-mediated neuromodulation of retinal synapses and the functional implications of such modulation in visual responses.

Early electrophysiological evidence from the cat and rabbit retinas demonstrated that exogenous application of either 5-HT or selective 5-HTR agonists and antagonists can modify light-evoked responses and spontaneous activity in some types of retinal ganglion cells (RGCs) [23–26]. More recently, in the mouse retina, the 5-HT2C receptor subtype has been suggested to be necessary for RGC responses to patterned visual stimuli [27] and in rats, 5-HT1AR has been shown to regulate both excitatory and inhibitory neurotransmitter release onto RGCs in a chronic glaucoma model [28, 29], further supporting the idea that 5-HT through different receptor subtypes can regulate RGC activity in the vertebrate retina. At synaptic sites, the activity of 5-HT and its receptors is tightly controlled by SERT, a plasma membrane transporter primarily responsible for reuptaking 5-HT from the synaptic cleft back into neurons and glia, thereby terminating the physiological action of 5-HT at the synapse [30]. In the vertebrate retina, SERT has been reported to be expressed in a subset of bipolar and amacrine cells that accumulate 5-HT [15] and in some RGCs [31, 32], where it has been suggested to play a role in the correct development of RGCs axonal projections [33]. However, the contribution of SERT to 5-HT-mediated modulation of synaptic function and visual response in adult RGCs remains unknown.

To address this question, we investigated how increasing 5-HT levels either by acute application of 5-HT or pharmacological blockade of SERT in mouse retinal slices impacts light-evoked excitatory response and spontaneous excitatory synaptic transmission in different types of RGCs. Altogether our results reveal a cell-type specific regulation by 5-HT, highlighting a presynaptic mechanism of action and the role of SERT in regulating excitatory synaptic strength in the inner retina.

## Material and methods

### Animals

Experiments were conducted using dim-light adapted retinal slices obtained from C57BL/6 J wild type (WT) and homozygous SERT knock-out (KO) mice [34] between postnatal day (P) 30 and P50 of either sex. Animals were housed at ~ 20 °C with ad libitum access to food and water on a 12:12 h light/dark cycle. All experimental procedures were performed in accordance with the bioethics regulations of the Chilean Research Council (ANID) and approved by the bioethics committee of the Universidad de Valparaíso, Chile (BEA159-20).

### Ex vivo* electrophysiology*

**Fig. 1:**
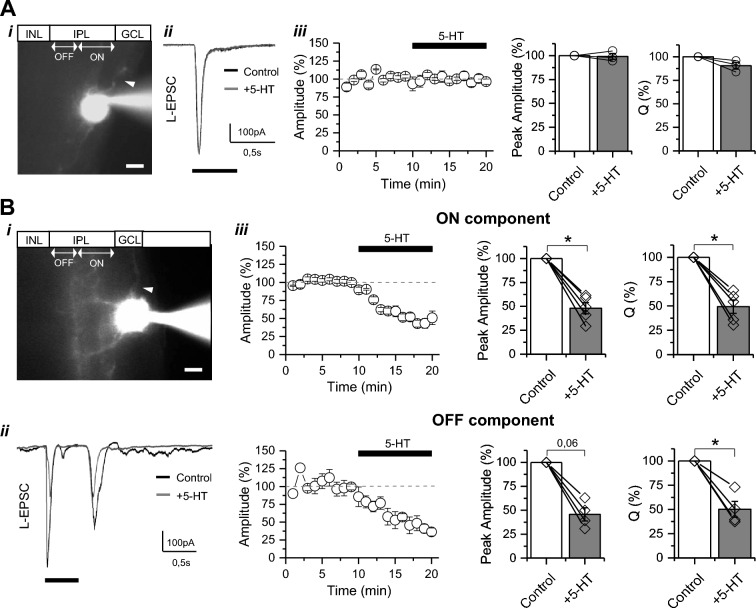
Exogenous serotonin reduces light-evoked response in ON–OFF but not in ON RGCs. **A** Effects of serotonin in ON RGC subtype. (i) Micrograph of a typical ON RGC filled with AlexaFluor488 (40x; scale bar = 10 µm; arrowhead indicates axon at the ganglion cell layer (GCL) border)**.** INL, inner nuclear layer; IPL, inner plexiform layer. (ii) Representative traces of the L-EPSC evoked by a 0,5 s light pulse (*black rectangle*) under basal conditions (control, *black*) and after bath application of 5-HT (50 µM) for 10 min (gray). (iii) Time course and summarized graphs showing no changes in amplitude and synaptic charge (Q) of L-EPSC evoked in ON RGCs following 5-HT application. **B** Effects of serotonin in ON–OFF RGC subtype. (i) Micrograph of a typical ON–OFF RGC (40x; scale bar = 10 µm; arrowhead indicates axon at the GCL border). (ii) Representative traces of the L-EPSC recorded in basal conditions (control, *black*) and after bath application of 5-HT (*gray*). (iii) Time course and summarized graphs showing the reduction induced by 5-HT in both the ON and OFF component of the L-EPSC. Statistical differences were assessed by paired t-test comparison of the means. *p < 0,05. (ON RGCs: n = 3 cells/3 animals, ON–OFF RGCs: n = 5 cells/5 animals)

Acute retinal slices (210 μm thick) were obtained using previously described methods [35–38]. Briefly, animals were euthanized following isoflurane anesthesia, eyes were enucleated, the cornea, lens and vitreous humor removed, and the retina isolated at room temperature (RT) in artificial cerebrospinal fluid (ACSF) composed by (in mM): 119 NaCl, 23 NaHCO_3_, 1,25 Na_2_HPO_4_, 2,5 KCl, 2,5 CaCl_2_, 1,5 MgSO_4_, 10 glucose, 2 Na^+^-pyruvate and 2 Na^+^-lactate (290–295 mOsm). ACSF was continuously bubbled with carbogen (95% O_2_/5% CO_2_) and the pH was adjusted to 7.4 with NaOH. Retinas were embedded in low-melting agar (3% p/v low-melting agarose in ACSF-HEPES, in mM: 119 NaCl, 24 HEPES, 1,25 Na_2_HPO_4_, 2,5 KCl, 2,5 CaCl_2_, 1,5 MgSO_4_, pH 7,4) and cut on a Leica VT1200S vibratome. Retinal slices were maintained for a 30 min stabilization period in ACSF before being moved to the recording chamber beneath a 40X water immersion lens on a fixed-stage Nikon FN1 upright microscope, perfused at a rate of 1–2 mL/min with ACSF at 29 ± 1 °C. For whole-cell patch-clamp recordings, RGCs (~ 80 µm deep) were visualized using infrared differential interference contrast and identified by the location of their somata in the ganglion cell layer. RGCs were discriminated from displaced amacrine cells for their axon, larger soma (diameter > 10 µm) and lower input resistance (< 600 MΩ) [39]. RGCs were differentiated by their response to light stimulation (see Fig. 1) and AlexaFluor-488 hydrazide (10 µM) was also added to the internal solution to confirm typical RGC morphology and to distinguish ON, OFF and ON–OFF RGC subtypes, based on dendritic stratification in the inner plexiform layer (IPL) [40]. All experiments were conducted in mesopic conditions, at a mean illuminance of 10 lx.

Voltage-clamp recordings were performed with patch electrodes (4.5–6 MΩ) containing (in mM): 90 Cs-methanesulfonate, 20 TEA (tetraethylammonium)-Cl, 10 HEPES, 10 EGTA, 10 Na2-phosphocreatine, 2 Mg-ATP, 0.3 Na-GTP, 0.01 AlexaFluor-488, adjusted to pH 7.35 with CsOH [39]. Spontaneous excitatory postsynaptic currents (sEPSCs) and light-evoked excitatory postsynaptic currents (L-EPSCs) were recorded at −60 mV in the continuous presence of picrotoxin (PTX, 50 µM), strychnine (STRY, 3 µM) and tetrodotoxin (TTX, 0,5 µM) to block GABA- and glycine-mediated inhibitory transmission and Na^+^ channels, respectively. L-EPSCs were evoked by a 500 ms light pulse (λ = 450 nm) delivered through the SOLA SE II light source (50% intensity) at an interval of 60 s, whereas pressure-induced release (“puff”) of L-glutamate (500 µM, 50 ms, 4–6 psi) in the IPL was used to elicit glutamate-induced currents in RGCs.

All currents were recorded using a Multiclamp 700B Amplifier (Molecular Devices), low pass filtered at 2 kHz and acquired at 10 kHz in a custom program written in Igor Pro 6.37 (WaveMetrics, Lake Oswego, USA). Series resistance was monitored continuously during the recording and cells with a variation greater than 20% were excluded from analysis. sEPSC recordings were analyzed offline using the event detection tool Mini Analysis Program (Synaptosoft). Traces were low-pass filtered at 3 kHz to improve the signal-to-noise ratio and the threshold amplitude for event detection was adjusted to ± 10 pA, above the double of the root mean square noise level (3–4 pA). Events were subsequently checked manually for accuracy. For L-EPSCs and glutamate-induced currents peak amplitude, synaptic charge (i.e. area under the curve) and decay time were analyzed with NeuroMatic [41], in Igor Pro 6.37.

### Statistical analysis

Unless otherwise indicated, data are presented as mean ± S.E.M, and statistical analysis was performed using Origin Pro 2018 (v9.5.1.195, OriginLab). To evaluate the effects of 5-HT (50 µM), and the selective 5-HT reuptake inhibitor citalopram (10 µM), application onto excitatory currents recorded in RGCs, a paired t-test was performed, comparing the last 5 min in the presence of the pharmacological agent with a 10 min control condition. Statistical significance was reached when p < 0.05. The number of cells, animals, and statistical tests used in each experiment are indicated in the figure legend.

## Results

### 5-HT reduces light-evoked response in RGCs in a cell-type specific manner

To evaluate the impact of increased extracellular levels of 5-HT on retinal synaptic function, we recorded light-evoked excitatory postsynaptic currents (L-EPSC) from RGCs (V_hold_ = − 60 mV) in acute mouse retinal slices. RGCs were classified into ON or ON–OFF subtypes based on their morphology and L-EPSC pattern (Fig. 1). While ON RGCs dendrites extend in the inner part of the IPL (Fig. 1A i) and exhibited an L-EPSC at the onset of light stimulation (Fig. 1A ii), ON–OFF RGCs have a bistratified dendritic field, expanding also in the outer layer of the IPL (Fig. 1B i), and display a L-EPSC at both the onset and offset of the light stimulus (Fig. 1B ii). After identifying the RGC type, we recorded the L-EPSC under basal conditions for at least 10 min and next evaluated the effect of bath application of 5-HT (50 µM, 10 min). While 5-HT had no effect on the amplitude and synaptic charge of the L-EPSC recorded from ON RGCs (Fig. 1A iii; Supplementary Table 1), it strongly reduced both the ON and OFF components of the L-EPSC recorded from ON–OFF RGCs (Fig. 1B iii; Supplementary Table 1), indicating a cell-type specific modulation of L-EPSC in mouse RGCs by 5-HT.

### Endogenous 5-HT also reduces L-EPSC in a cell-type specific manner

**Fig. 2:**
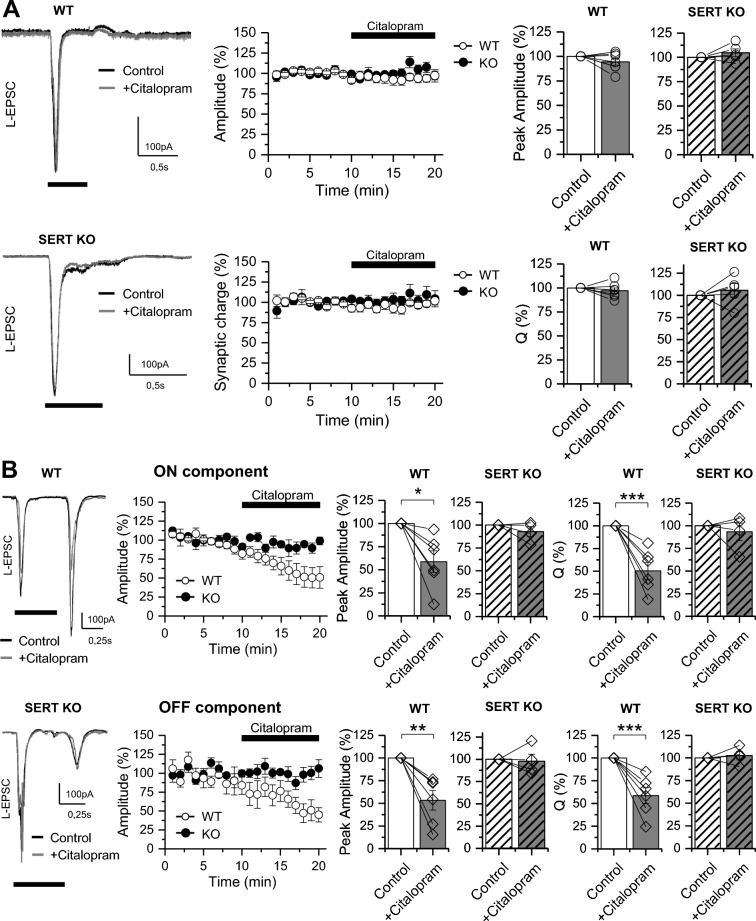
Endogenous serotonin reduces L-EPSC in ON–OFF but not in ON RGCs. **A** *Left:* Representative traces of the L-EPSCs evoked by a 0,5 s light pulse (*black rectangle*) in ON RGCs under basal conditions (control; *black*) and 10 min after bath application of citalopram (10 µM; *gray*) in wild type (WT; *upper panels*) and SERT KO retinas (*lower panels*). *Right:* Summarized graphs showing that citalopram had no effect on the amplitude and synaptic charge of L-EPSC in ON RGCs from WT and SERT KO mice. **B** *Left:* Representative traces of the L-EPSCs evoked by a 0,5 s light pulse (black rectangle) in ON–OFF RGCs in basal conditions (control; *black*) and 10 min after bath application of 10 µM citalopram (gray) in both WT (*upper traces*) and SERT KO retinas (*lower traces*). *Right:* Summary plots showing the reduction induced by citalopram in the ON (*top panels*) and OFF component (*bottom panels*) of the L-EPSC recorded from WT retinas, an effect that was absent in SERT KO retinas. Statistical differences were assessed by paired t-test comparison of the means. *p < 0,05; **p < 0,01; ***p < 0,001. (ON RGCs: WT n = 7 cells /6 animals; SERT KO: n = 5 cells/5 animals; ON–OFF RGCs: WT n = 6 cells/6 animals and SERT KO n = 4 cells/4 animals)

To further determine whether 5-HT-mediated effect on L-EPSC is inducible by an endogenous increase in the tone of 5-HT in retinal slice, we bath applied the 5-HT reuptake inhibitor citalopram (10 μM) for 10 min while L-EPSC were evoked in ON and ON–OFF RGCs (Fig. 2). While citalopram had no effect on the amplitude or synaptic charge of L-EPSC in ON RGCs (Fig. 2A), it significantly reduced both components of the L-EPSC in ON–OFF RGCs (Fig. 2B; Supplementary Table 1). To confirm that citalopram effect on L-EPSC was mediated by 5-HT reuptake blockade and the consequent increase in 5-HT extracellular levels, we recorded L-EPSC in RGCs from null SERT mice retinas (SERT KO; Fig. 2A, B). While typical L-EPSC patterns were elicited in both ON and ON–OFF RGCs from SERT KO retina (Fig. 2A, B), these responses remained unaltered after bath application of citalopram (Fig. 2A, B; Supplementary Table 1), further confirming that citalopram-induced depression of L-EPSCs is entirely mediated through inhibition of SERT. Moreover, these results indicate that by controlling the levels of 5-HT in the retina, SERT impacts RGCs activity in a cell-type specific manner.

### 5-HT-mediated reduction of excitatory synaptic transmission onto ON–OFF RGCs is likely presynaptic

**Fig. 3:**
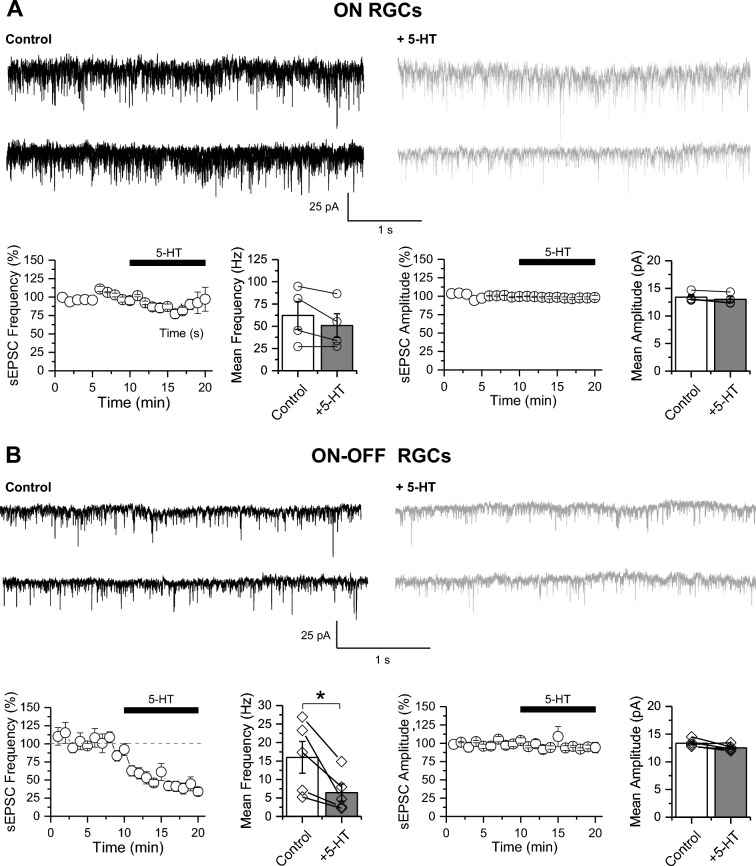
Serotonin reduces the frequency of spontaneous excitatory synaptic transmission onto ON–OFF RGCs. **A** Effects of serotonin in ON RGC subtype. *Top*: Representative traces of the sEPSCs recorded in ON RGCs under basal conditions (control, black, *left*) and 10 min after bath application of 5-HT (50 µM; gray, *right*). *Bottom:* Summarized graphs showing the effect of 5-HT on the sEPSC frequency (*left*) and amplitude (*right*). No statistical differences were detected by paired t-test comparison of the means (n = 4 cells/4 animals). **B** Effects of serotonin in ON–OFF RGC subtype. *Top:* Representative traces of the sEPSCs recorded in ON–OFF RGCs in basal conditions (control, black, *left*) and 10 min after bath application of 5-HT (50 µM; gray, *right*). *Bottom:* Summarized graphs showing the effect of 5-HT on the frequency (*left*) but not the amplitude (*right*) of sEPSC. *p < 0,05 (n = 5 cells /4 animals)

**Fig. 4:**
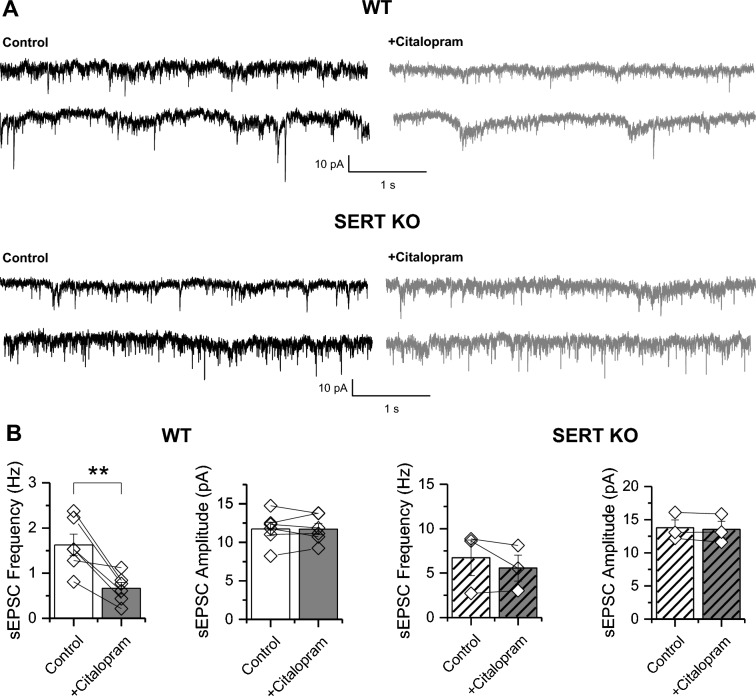
Endogenous serotonin reduces the frequency of spontaneous excitatory synaptic transmission onto ON–OFF RGCs. **A** Representative traces of the sEPSCs recorded in ON–OFF RGCs under basal conditions (control, black, *left*) and 10 min after bath application of citalopram (10 µM; gray; *right*) in wild type (WT; *upper traces*) and in SERT KO retina (*lower traces*). **B** Summarized graphs showing the significant changes in the frequency (**p < 0.01) but not in amplitude of the sEPSC recorded from WT retina (*left*) after bath application of citalopram. Note that citalopram had no effect on the frequency or amplitude of sEPSC recorded from ON–OFF RGCs in SERT KO retinas (*right*). Statistical differences were assessed by paired t-test comparison of the means. (WT n = 6 cells/6 animals; SERT KO n = 3 cells/3 animals)

To further evaluate the potential cellular mechanism underlying this cell-type specific reduction of L-EPSC by 5-HT, we next recorded isolated (*see methods*) spontaneous excitatory postsynaptic currents (sEPSC) from ON and ON–OFF RGCs under basal conditions and after bath application of 5-HT (Fig. 3). Consistent with the cell-type specific effect mediated by 5-HT (Figs. 1, 2), we found that 5-HT had no effect on the frequency neither in the amplitude of sEPSCs in ON RGCs (Fig. 3A; Supplementary Table 1), but significantly reduced the frequency without altering the amplitude of sEPSCs in ON–OFF RGCs (Fig. 3B; Supplementary Table 1). This change in the frequency of sEPSC suggests a presynaptic mechanism of action regulating excitatory neurotransmitter release onto ON–OFF RGCs. Similarly, we found that an endogenous increase in the tone of 5-HT by bath application of citalopram also reduced the frequency of sEPSCs in ON–OFF RGCs without affecting their amplitude (Fig. 4A; Supplementary Table 1), suggesting a presynaptic mechanism of action. Importantly, citalopram-mediated reduction in the frequency of sEPSC was absent in ON–OFF RGCs recorded from SERT KO retinas (Fig. 4B; Supplementary Table 1), further demonstrating a role of SERT and 5-HT tone in regulating excitatory synapses onto ON–OFF RGCs in the inner retina.

**Fig. 5:**
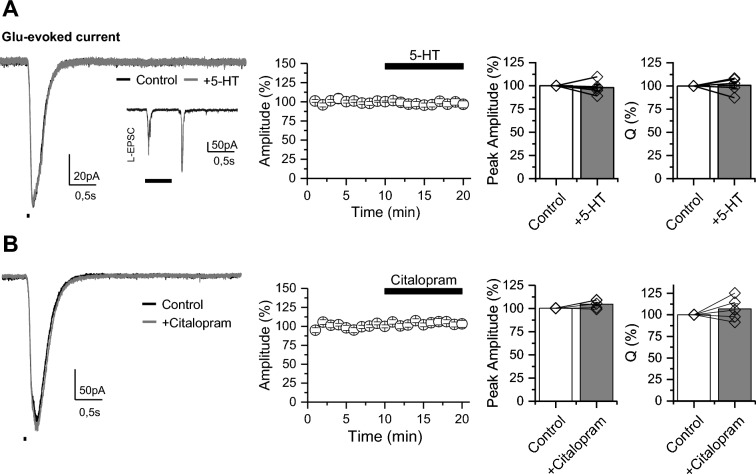
Serotonin has no effect on glutamate-evoked currents in ON–OFF RGCs. **A** 5-HT (50 µM) did not affect glutamate-evoked response in ON–OFF RGCs elicited by 50 ms puff (*black rectangle*) of 500 µM L-glutamate (Glu) delivered in the IPL. *Left*: Representative traces recorded under basal conditions (control, *black*) and 10 min after bath application of 5-HT (*gray*). Inset shows a typical L-EPSC evoked, used to confirm the ON–OFF RGC identity. *Right*: Time course and summary plots showing the effect of 5-HT on glutamate-evoked response (n = 4 cells/3 animals). **B** Representative traces (left) and summarized graphs (right) showing that citalopram (10 µM) did not affect glutamate-evoked response in ON–OFF RGCs. No statistical differences were detected by paired t-test comparison of the means (n = 5 cells/2 animals)

To further confirm the presynaptic origin in the 5-HT- and citalopram-mediated reduction of excitatory synaptic strength onto ON–OFF RGCs, we bypassed excitatory neurotransmitter release by directly activating postsynaptic glutamate receptors on RGC using focal pressure application of L-glutamate in the IPL (Fig. 5). Under this experimental condition, no changes in the amplitude or charge transfer were found in ON–OFF RGCs upon application of 5-HT (Fig. 5A; Supplementary Table 1) or citalopram (Fig. 5B; Supplementary Table 1). Altogether, these results reveal that exogenous and endogenous 5-HT depress excitatory synaptic transmission onto RGCs, in a cell-type specific manner, highlighting a potential presynaptic mechanism of action and a role of SERT in regulating 5-HT-mediated effect onto ON–OFF RGCs.

## Discussion

Previous evidence indicates that the serotonergic system is implicated in the regulation of mammalian RGC function at various levels, including modulation of RGC activity [23–26], developmental influences [27, 32, 33] and neuroprotective roles [7, 28, 29], all of which are crucial for maintaining retinal function and visual processing. Accordingly, our study reveals that exogenous and endogenous increase in the levels of 5-HT produces a cell-type specific effect in mouse retina, impacting excitatory synaptic strength of both spontaneous and light-evoked currents in ON–OFF, but not in ON RGCs. Although RGCs is a very heterogenous group of cells [42, 43], an observation that could explain the inherent variability of spontaneous activity across experiments (see Figs. 3B and 4B), our results clearly demonstrated a homogeneous effect of both 5-HT (Fig. 3B) and citalopram (Fig. 4B) to reduce excitatory inputs onto ON–OFF RGCs. Moreover, our data suggest that 5-HT-mediated reduction of excitatory synaptic strength is likely to be presynaptic (Figs. 3, 4 and 5), open the possibility that activation of presynaptic 5-HTRs that through different canonical signaling pathways, including decrease of cAMP levels, activation of GIRK-mediated hyperpolarization or by regulate voltage-gated calcium channels [44], could regulate neurotransmitter release at either photoreceptor terminals in the outer retina or at bipolar cell (BC) terminals in the inner retina. Although our electrophysiological study does not directly identify the specific 5-HTRs subtypes involved in this selective modulation, it does provide evidence that SERT, as in many brain synapses [45], controls 5-HT-mediated effects on RGCs likely by regulating excitatory presynaptic neurotransmitter release. These findings highlight the crucial role of 5-HT and SERT in controlling retinal visual processing at the RGC level.

Increasing evidence points out that long-term treatment with selective 5-HT reuptake inhibitors (SSRIs), that effectively block 5-HT reuptake by blocking SERT and enhance 5-HT tone, can produce a range of adverse effects on vision including reduced visual acuity, night blindness, glaucoma and optic neuropathy [46, 47]. Although pharmacological agents, including citalopram and other SSRIs have recently been shown to act also at the neurotrophin receptor TrKB (Tropomyosin receptor kinase B) [48], our results using SERT deficient retinas (Figs. 2 and 4) strongly support that citalopram effect on light-evoked response and synaptic function is likely mediated through the blockade of SERT rather than TrkB receptors. Moreover, these results also suggest that alternative transporters, like organic cation transporters that could mediate 5-HT reuptake and some of the effect of SSRIs in the absence of SERT [49–51], are unlikely to play a role in regulating RGCs activity. While the exact distribution of SERT within the retina is still unclear, the cell-type specific effect of 5-HT (Fig. 1) and citalopram (Fig. 2) suggests that it might be expressed by neurons within the ON–OFF pathway. It is also possible that SERT, which is known to be expressed in RGCs during development [31, 33], is located on ON–OFF RGCs dendrites and, therefore, throughout all subfields of the inner retina, where it may contribute to shape visual processing at retinal level. Further experiments are required to unravel the precise localization of SERT and its impact on regulating retinal 5-HT tone to modulate retinal synaptic function.

### 5-HT-mediated effects in mouse RGCs are cell-type specific

Early evidence demonstrated that 5-HT suppresses both the spontaneous activity and light-evoked discharge of ON-center RGCs and enhances the activity of OFF-center RGCs in cat retina [26]. Likewise, in the rabbit retina, bath application of 5-HT1A agonist or 5-HT2Rs antagonist reportedly reduces ON RGC response [24, 25], whereas 5-HT3 agonist increases ON responses [23]. While this evidence suggests that 5-HT or different 5-HTRs agents can regulate ON RGCs, our results in mouse retina reveal that 5-HT induces a cell-type specific depression of excitatory synaptic inputs and light-evoked response impacting selectively ON–OFF but not ON RGCs. While these differences could be due to the animal model and/or method used to evaluate 5-HT mediated effects in acute retinal slices, we found that the ON component of the ON–OFF RGCs response was strongly reduced by increasing levels of 5-HT, suggesting that some type of ON BC that connect to ON–OFF RGCs, are different from those ON BC that make synaptic contact onto ON RGCs, and are sensitive to 5-HT. While multiple bipolar cell types converge onto a single RGC to convey different visual signals [52], distinct RGCs are contacted in different proportion by specific BC types [53]. For example, BC5R type has been shown to convey the ON signal mainly to RGCs of the ON–OFF class, while BC6 preferentially contacts ON RGCs, including the ON sustained (ON-s) alpha type and the intrinsic photosensitive RGCs [53–56]. OFF-s and OFF-t alpha RGCs, on the other side, mainly receive excitatory synapses from OFF BC2 and 4, respectively, though they are contacted by all five OFF BC types [57]. Therefore, it is possible that just the inputs from ON BCs that preferentially target ON–OFF RGCs, like BC5R, might be specifically regulated by 5-HT. It could also be possible that different synaptic boutons of the same BC are differentially regulated by 5-HT, providing a synapse-specific rather than a cell type-specific regulation. Additionally, by regulating synaptic function between photoreceptor and BCs in the outer retina, 5-HT could also impact RGC response by regulating specific BC activity. Adding to this complexity, it is noteworthy that, in our experimental conditions the effects of 5-HT and citalopram were studied in the presence of GABA and glycine receptor blockers, which enhance glutamate release. As 5-HT reportedly regulate inhibitory inputs in rat retina [28, 29], whether this cell-type specific effect of 5-HT could be also observed in the absence of inhibitory blockers remains to be assessed. Further studies are required to test these possibilities and to clarify the exact mechanism underlying 5-HT-mediated effects at excitatory retinal synapses, including the 5-HTR subtype(s) involved in this modulation.

In summary, our results in retinal circuitry demonstrated that the regulation of visual processing by 5-HT can occur at all visual sensory stations. Besides its action in the visual cortex [58–61], where the integration of visual information occurs, recent evidence reported that 5-HT can also regulate RGC inputs to the thalamus [62] and our present results using acute retinal slices demonstrated that 5-HT, and in particular, SERT are important modulators of retinal synaptic function, ultimately indicating that the serotonergic system is transversally implicated in the neuromodulation of visual processing. Further experiments would be helpful to clarify how and under which circumstances 5-HT can control vision in health and disease.

## Supplementary Information

Supplementary Material 1. Table 1. Quantitative and statistical analysis related to Figures 1 to 5

## Data Availability

The datasets used and/or analyzed during the current study are available from the corresponding author on reasonable request.
