# Supplementary material for: Serotonin regulates in a cell-type specific manner light-evoked response and synaptic activity in mouse retinal ganglion cells
Source: Biol Res. 2025 Mar 4;58:11. doi: 10.1186/s40659-025-00594-6 (PMC11877958; doi:10.1186/s40659-025-00594-6)
Supplement: Supplementary file 1 — Supplementary Material 1. Table 1. Quantitative and statistical analysis related to Figures 1 to 5 [file 40659_2025_594_MOESM1_ESM.docx]

**Supplementary table1. Quantitative and statistical analysis related to Figures 1 to 5.**

|  | RGC type | Control  (mean ± S.E.M) | Treatment  (mean ± S.E.M) | n  cells/animals (c/a) | p value  (paired t test) |
| --- | --- | --- | --- | --- | --- |
| Figure 1A |  | | **5-HT 50 µM** |  |  |
| L-EPSC amplitude (pA) | ON | 419,98 ± 195,46 | 421,93 ± 193,98 | 3c/3a | 0,8769 |
| Normalized on control (%) |  | 100 | 99,21 ± 3,01 |  |  |
| L-EPSC synaptic charge (pA*s) | ON | 79,29 ± 48,17 | 73,27 ± 44,18 | 3c/3a | 0,2813 |
| Normalized on control (%) |  | 100 | 90,49 ± 3,55 |  |  |
| Figure 1B – ON component |  |  |  |  |  |
| L-EPSC amplitude (pA) | ON-OFF | 448,89 ± 101,87 | 206,17 ± 47,39 | 5c/5a | 0,0195 |
| Normalized on control (%) |  | 100 | 48,07 ± 5,94 |  |  |
| L-EPSC synaptic charge (pA*s) | ON-OFF | 70,32 ± 12,38 | 19,10 ± 6,93 | 5c/5a | 0,0275 |
| Normalized on control (%) |  | 100 | 49,34 ± 6,90 |  |  |
| Figure 1B – OFF component |  |  |  |  |  |
| L-EPSC amplitude (pA) | ON-OFF | 370,32 ± 125,13 | 174,50 ± 67,35 | 4c/4a | 0,0572 |
| Normalized on control (%) |  | 100 | 45,69 ± 6,98 |  |  |
| L-EPSC synaptic charge (pA*s) | ON-OFF | 35,65 ± 9,21 | 18,10 ± 5,28 | 4c/4a | 0,0382 |
| Normalized on control (%) |  | 100 | 50,15 ± 8,18 |  |  |
| Figure 2A - WT mice |  | | **Citalopram 10** **µM** |  |  |
| L-EPSC amplitude (pA) | ON | 356,95 ± 68,69 | 333,13 ± 60,37 | 7c/6a | 0,1903 |
| Normalized on control (%) |  | 100 | 94,42 ± 3,46 |  |  |
| L-EPSC synaptic charge (pA*s) | ON | 29,13 ± 5,97 | 28,26 ± 5,61 | 7c/6a | 0,3464 |
| Normalized on control (%) |  | 100 | 97,16 ± 2,91 |  |  |
| Figure 2A - SERT KO mice |  |  |  |  |  |
| L-EPSC amplitude (pA) | ON | 284,27 ± 124,85 | 285,11 ± 117,04 | 5c/5a | 0,9530 |
| Normalized on control (%) |  | 100 | 104,68 ± 3,96 |  |  |
| L-EPSC synaptic charge (pA*s) | ON | 27,35 ± 11,36 | 26,08 ± 8,65 | 5c/5a | 0,7170 |
| Normalized on control (%) |  | 100 | 105,83 ± 7,61 |  |  |
| Figure 2B - WT mice  ON component |  |  |  |  |  |
| L-EPSC amplitude (pA) | ON-OFF | 388,71 ± 89,00 | 252,58 ± 82,08 | 6c/6a | 0,0148 |
| Normalized on control (%) |  | 100 | 58,71 ± 11,67 |  |  |
| L-EPSC synaptic charge (pA*s) | ON-OFF | 24,46 ± 5,13 | 13,76 ± 4,06 | 6c/6a | 0,0049 |
| Normalized on control (%) |  | 100 | 50,53 ± 9,31 |  |  |
| Figure 2B - WT mice  OFF component |  |  |  |  |  |
| L-EPSC amplitude (pA) | ON-OFF | 545,25 ± 129,02 | 345,76 ± 97,92 | 6c/6a | 0,0088 |
| Normalized on control (%) |  | 100 | 53,29 ± 10,72 |  |  |
| L-EPSC synaptic charge (pA*s) | ON-OFF | 53,14 ± 15,21 | 37,38 ± 13,66 | 6c/6a | 0,0019 |
| Normalized on control (%) |  | 100 | 58,63 ± 8,79 |  |  |
| Figure 2B - SERT KO mice  ON component |  |  |  |  |  |
| L-EPSC amplitude (pA) | ON-OFF | 206,63 ± 147,69 | 200,32 ± 149,19 | 4c/4a | 0,3216 |
| Normalized on control (%) |  | 100 | 92,96 ± 5,39 |  |  |
| L-EPSC synaptic charge (pA*s) | ON-OFF | 11,73 ± 7,66 | 10,76 ± 7,33 | 4c/4a | 0,2441 |
| Normalized on control (%) |  | 100 | 93,28 ± 9,65 |  |  |
| Figure 2B - SERT KO mice  OFF component |  |  |  |  |  |
| L-EPSC amplitude (pA) | ON-OFF | 206,37 ± 72,86 | 185,94 ± 60,75 | 4c/4a | 0,2546 |
| Normalized on control (%) |  | 100 | 97,80 ± 7,86 |  |  |
| L-EPSC synaptic charge (pA*s) | ON-OFF | 16,15 ± 5,02 | 16,23 ± 5,15 | 4c/4a | 0,8888 |
| Normalized on control (%) |  | 100 | 102,61 ± 4,68 |  |  |
| Figure 3A |  | | **5-HT 50µM** |  |  |
| sEPSC frequency (Hz) | ON | 62,10 ± 15,67 | 50,74 ± 13,31 | 4c/4a | 0,1282 |
| Normalized on control (%) |  | 100 | 83,78 ± 7,53 |  |  |
| sEPSC amplitude (pA) | ON | 13,40 ± 0,43 | 13,00 ± 0,47 | 4c/4a | 0,1231 |
| Normalized on control (%) |  | 100 | 96,98 ± 1,47 |  |  |
| Figure 3B |  |  |  |  |  |
| sEPSC frequency (Hz) | ON-OFF | 15,98 ± 4,29 | 6,46 ± 2,33 | 5c/5a | 0,0274 |
| Normalized on control (%) |  | 100 | 40,02 ± 5,85 |  |  |
| sEPSC amplitude (pA) | ON-OFF | 13,34 ± 0,31 | 12,53 ± 0,26 | 5c/5a | 0,0811 |
| Normalized on control (%) |  | 100 | 94,08 ± 2,47 |  |  |
| Figure 4B - WT mice |  | | **Citalopram 10** **µM** |  |  |
| sEPSC frequency (Hz) | ON-OFF | 1,63 ± 0,24 | 0,67 ± 0,13 | 6c/6a | 0,0065 |
| Normalized on control (%) |  | 100 | 42,07 ± 9,30 |  |  |
| sEPSC amplitude (pA) | ON-OFF | 11,75 ± 0,87 | 11,74 ± 0,73 | 6c/6a | 0,9802 |
| Normalized on control (%) |  | 100 | 100,76 ± 3,66 |  |  |
| Figure 4B – SERT KO mice |  |  |  |  |  |
| sEPSC frequency (Hz) | ON-OFF | 6,72 ± 2,01 | 5,56 ± 1,47 | 3c/3a | 0,3658 |
| Normalized on control (%) |  | 100 | 89,26 ± 13,73 |  |  |
| sEPSC amplitude (pA) | ON-OFF | 13,78 ± 1,18 | 13,53 ± 1,24 | 3c/3a | 0,3068 |
| Normalized on control (%) |  | 100 | 98,09 ± 1,52 |  |  |
| Figure 5A |  | | **5-HT 50µM** |  |  |
| Glu puff-evoked current –  peak amplitude (pA) | ON | 382,38 ± 134,51 | 367,10 ± 127,23 | 5c/4a | 0,2926 |
| Normalized on control (%) |  | 100 | 98,02 ± 3,39 |  |  |
| Glu puff-evoked current –  synaptic charge (pA*s) | ON | 151,13 ± 48,88 | 146,56 ± 44,56 | 5c/4a | 0,6072 |
| Normalized on control (%) |  | 100 | 100,83 ± 3,78 |  |  |
| Figure 5B |  | | **Citalopram 10** **µM** |  |  |
| Glu puff-evoked current –  peak amplitude (pA) | ON-OFF | 175,46 ± 41,81 | 185,92 ± 45,71 | 5c/2a | 0,0826 |
| Normalized on control (%) |  | 100 | 104,65 ± 2,05 |  |  |
| Glu puff-evoked current –  synaptic charge (pA*s) | ON-OFF | 156,21 ± 28,93 | 173,36 ± 41,74 | 5c/2a | 0,2687 |
| Normalized on control (%) |  | 100 | 106,86 ± 5,97 |  |  |
